# Trend in H_2_S Biology and Medicine Research—A Bibliometric Analysis

**DOI:** 10.3390/molecules22122087

**Published:** 2017-11-29

**Authors:** Guangdong Yang, Lingyun Wu

**Affiliations:** 1 Department of Chemistry and Biochemistry, Laurentian University, 935 Ramsey Lake Road, Sudbury, ON P3E 2C6, Canada; 2 Cardiovascular and Metabolic Research Unit, Laurentian University, 935 Ramsey Lake Road, Sudbury, ON P3E 2C6, Canada; 3 School of Human Kinetics, Laurentian University, 935 Ramsey Lake Road, Sudbury, ON P3E 2C6, Canada; 4 Health Science North Research Institute, Sudbury, ON, P3E 5J1, Canada

**Keywords:** H_2_S, biology and medicine, bibliometric, web of science, *h*-index

## Abstract

The biological and medical importance of hydrogen sulfide (H_2_S) has been recognized for decades. The aim of this bibliometric study is to analyze the quantity and quality of publications in H_2_S biology and medicine (H_2_SBM) based on the databases of Web of Science and Google Scholar. A total of 5881 publications published between 1990 and 2016 were analyzed. The number of H_2_SBM papers published before 2004 was below 100 annually, but thereafter this number rapidly increased and peaked in 2015 with more than 7-fold increase. All publications related to H_2_SBM research achieved a total *h*-index of 136 and were cited 123,074 times. The most published disciplines in H_2_S biomedicine research were the cardiovascular system (8.5%), neuroscience (6.5%), and gastroenterology hepatology (4.7%). The country with the greatest number of publications in the H_2_SBM research field was the USA with 1765 (30.0%) publications, followed by China with 995 (16.9%) publications and Japan with 555 (9.4%) publications. The top 3 most published institutes were National University of Singapore, Peking University in China, and University of Groningen in Netherlands. Nitric Oxide Biology and Chemistry was the most exploited journal for H_2_SBM publications with 461 articles, followed by FASEB Journal with 200 publications and Antioxidants Redox Signaling with 116 publications. The most highly cited publications and researchers in H_2_SBM research were also unmasked from this bibliometric analysis. Collectively, H_2_SBM publications exhibit a continuous trend of increase, reflecting the increased H_2_SBM research intensity and diversity globally.

## 1. Introduction

Hydrogen sulfide (H_2_S) was historically considered to be highly toxic and hazardous to the environment. However, in recent decades, H_2_S has been recognized as a novel gasotransmitter, similar to nitric oxide and carbon monoxide [1,2,3,4,5,6,7,8,9,10]. The concept of gasotransmitter and the classifying criteria were first framed in 2002, and since then, we have witnessed the rapid growth of the research field in H_2_S biology and medicine (H_2_SBM) [2,11]. A large amount of work has been conducted globally leading to many breakthrough discoveries on the paramount roles of H_2_S in biology and medicine. H_2_S acts as a universal molecule in different species, including human, mouse, rat, plant, bacteria, virus, and many others [8]. Four international conferences on the Biology and Medicine of H_2_S have been held in Shanghai (China, 2009), Atlanta, GA (USA, 2012), Kyoto (Japan, 2014), and Naples (Italy, 2016) [12,13,14], and the 5th international conference will be held in Toronto, ON (Canada) in 2018. In addition, specific H_2_S-related topics or sections have been featured in numerous symposia such as European Conference on the Biology of Hydrogen Sulfide, and Experimental Biology Meeting. A unique organization, the European Network on Gasotransmitters, was formed in 2011. We are experiencing one of the most expanding research evolutions in the recent history of biomedical sciences.

Despite the large amount of H_2_SBM-related literature that has been published, a systematic analysis of the scientific literature in H_2_SBM research has not been conducted to date. Given the enormous number of publications, a bibliometric analysis that identifies comprehensively the major research topics and outcomes in H_2_SBM will provide insight and guidance for scientists and all relevant stakeholders of academia, industry, and health management. Bibliometrics is often used to measure and compare the publications within a given topic, field, journal, institute, funding agency or country, which would enable the comprehensive recognition of the most important and relevant scientific values and impact [15,16]. To this end, we undertook the present bibliometric study to explore the characteristics of global research publication output from H_2_SBM research by using publicly available databases including Web of Science and Google Scholar. 

## 2. Methods

### 2.1. Search Tools

The H_2_SBM data published between 1990 and 2016 were collected from the Thomson Scientific Web of Science core collection (http://www.webofknowledge.com/WOS), a multidisciplinary index to the journal literature of science and technology [17]. To gather the citation data for H_2_SBM papers, both Web of Science and Google Scholar databases were used up to 15 October 2017. Web of Science produces interdisciplinary literature databases, including the creditable journals of numerous research disciplines. It should be noted that Web of Science only represents about 15% of the total number of English language journals currently published worldwide [17]. In contrast, Google Scholar includes a wider variety of publications more than Web of Science does [18]. Although both Web of Science and Google Scholar have the powerful feature of tracking citing items, the times cited differ greatly between Web of Science and Google Scholar based on different searching algorithm.

### 2.2. Search Strategies

“H_2_S” or “hydrogen sulfide” or “hydrogen sulphide” was used as phrase to search topics with the mode of “basic search” of the Core collection of Web of Science from 1990 to 2016. This first search produced 37,921 articles, which include all the publications in H_2_S-related research disciplines. The Web of Science subject categories were further selected to search H_2_SBM articles, including physiology, biology, agricultural multidisciplinary, neuroscience, dentistry oral surgery medicine, immunology, respiratory system, critical care medicine, biochemistry, molecular biology, urology nephrology, hematology, zoology, clinical neurology, endocrinology metabolism, peripheral vascular disease, genetics heredity, plant science, oncology, medicine general internal, medicine research experimental, cell biology, gastroenterology hepatology, horticulture, cardiac cardiovascular systems, pharmacology pharmacy, surgery, and pathology. Meanwhile, the following subject categories were excluded: chemistry physical, engineering chemical, materials science multidisciplinary, chemistry multidisciplinary, environmental science, energy fuels, engineering environmental, electrochemistry, physical applied, geochemistry geophysics, metallurgy metallurgical engineering, physical atomic molecular chemical, biotechnology applied microbiology, chemistry organic, chemistry inorganic nuclear, microbiology, nanoscience nanotechnology, chemistry applied, physical condensed matter, geosciences multidisciplinary, water recourses, instrument instrumentation, multidisciplinary science, food science technology, engineering petroleum, thermodynamics, marine freshwater biology, oceanography, engineering electronic, mineralogy, geology, spectroscopy, toxicology, engineering mechanical, meteorology atmospheric science, ecology, optics, engineering civil, crystallography, mining mineral processing, green sustainable science technology, biophysical, public environmental occupational health, limnology, agricultural dairy animal science, material science ceramics, mechanics, material science paper wood, nuclear science technology, soil science, fisheries, engineering manufacturing, agronomy, computer science interdisciplinary applications, material science textiles, paleontology, construction building technology, remote sensing, engineering industrial, materials science biomaterials, and engineering biomedical. The inclusion and exclusion categories used in our study are intended to relatively focus on the publication directly related to H_2_SBM, not those with the focus on environment production of H_2_S, H_2_S-induced pollution, or H_2_S toxicology. In this study, two indicators for citation impacts are used, including the impact factor (IF) of journals in 2016 and the *h*-index. IF is a useful indicator to assess the citation quantity of journals [19]; and the *h*-index is used to measure the productivity and impact of published works from different researchers, research field, countries, institutes, or journals [20]. SSPS software 16.0 (Armonk, NY, USA) was used to analyze the groups’ correlation coefficient. 

## 3. Results

### 3.1. Evolution of H_2_SBM Research

**Figure 1:**
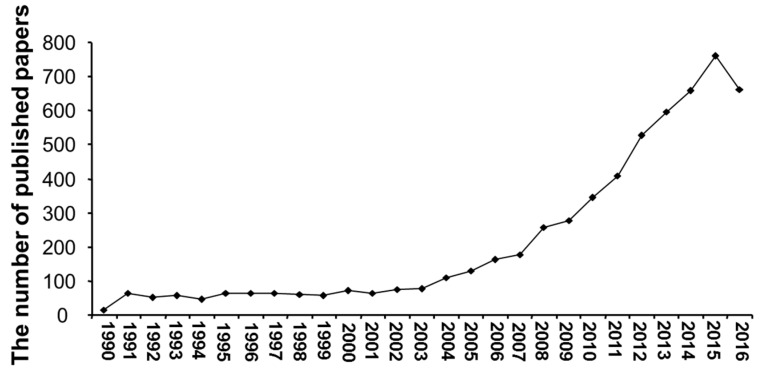
The number of H_2_SBM research publications between 1990 and 2016.

After applying the inclusion and exclusion criteria to the publication databases, 5881 publications in H_2_SBM research were registered from Web of Science core collection database. In recent decades, the literature on H_2_SBM has an overall increasing trend in the total number of scientific publications from 1990 to 2016 (Figure 1). The global evolution of H_2_SBM literature can be split into 2 phases. From 1990 to 2003, the H_2_SBM scientific literature was in a steady-state growth (from 13 publications in 1990 to 77 in 2003). Statistical analysis revealed a low positive significant correlation between the years during 1990 to 2003 and number of scientific publications (*r* = 0.74). The second phase was between 2004 and 2016, in which the quantity of publications was rapidly increased with the peak reached 760 publications in 2015. Among the 5881 publications, most (5060; 86.0%) were published after 2003 with an annual median growth rate of 56.9 articles per year. The publication number in 2016 was 13.0% less in comparison with that in 2015, the first year in which H_2_SBM publications were decreased. A positive significant correlation was found between the years (2004–2016) and number of scientific publications (*r* = 0.91).

### 3.2. The Top 10 Mostly Cited Publications in H_2_SBM Research

For all publications related to H_2_SBM research, an *h*-index of 136 and total citation of 123,074 times were generated (Web of Science core collection). The median number of citations per article is 20.9. The numbers of citations for the top 10 mostly cited articles ranged from 1073 to 456 by Web of Science or 1754 to 697 by Google Scholar (Table 1) [1,2,3,4,5,6,7,8,9,10]. The median number of citations for these top 10 mostly cited publications was 782 from Web of Science and 1168 from Google Scholar. The rank of the top 10 mostly cited publications listed in Table 1 was based on the average of the numbers of citations quoted from Web of Science and Google Scholar. The top 10 mostly cited articles were published between 1996 and 2012 in 9 different high-quality journals, including EMBO Journal, Science, FASEB Journal, Journal of Neuroscience, Nature Review Drug Discovery, Biochemical and Biophysical Research Communication, Proceedings of the National Academy of Sciences of the United States of America, Annual Review of Pharmacology and Toxicology, and Physiological Reviews. FASEB Journal published 2 top-cited articles (2nd and 10th). The impact factors for journals that published these top 10 mostly cited articles ranged from 57.0 (Nature Review Drug Discovery) to 2.47 (Biochemical and Biophysical Research Communication) in 2016.

There were 4 different countries of origin for the top 10 mostly cited articles. Canada had the largest number of articles, with 4 from the same group led by R Wang (1st, 2nd, 4th, and 8th). Japan produced 3 articles all with H Kimura as corresponding author (3rd, 6th, and 10th). USA contributed 2 articles, one being from C Szabo group (5th) and another from DJ Lefer group (7th). The 9th one was from PK Moore group in England. In these top 10 mostly cited articles, 6 articles (1st, 3rd, 4th, 6th, 7th, and 10th) were original research articles with 810 mean citation each (Web of science), and the other 4 were review or opinion articles with 741 mean citation each (Web of science). For those 6 research articles, 4 (1st, 4th, 6th, and 7th) were from cardiovascular research and 2 (3rd and 10th) were in neuroscience field.

### 3.3. The Most Receptive H_2_SBM Research Publication Avenues

**Figure 2:**
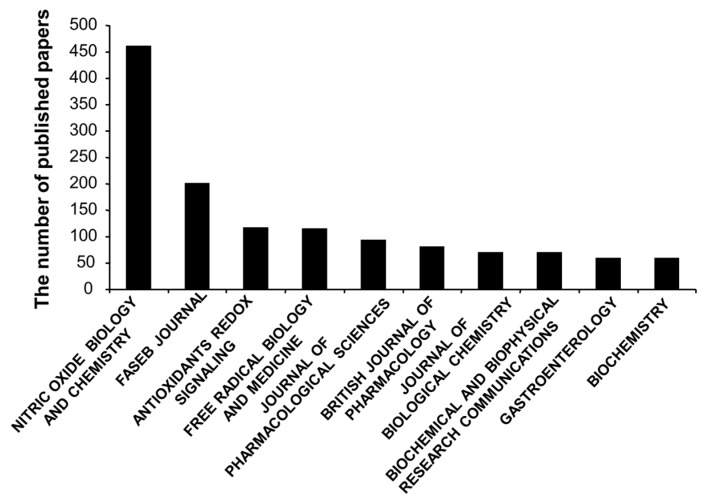
The most receptive journals for H_2_SBM research publication.

In total, 5881 H_2_SBM articles were published in a range of more than 100 different journals, and about 23.4% of the H_2_SBM related publications were published in the top 10 journals as listed in Figure 2. It is not surprised that Nitic Oxide Biology and Chemistry is the most receptive journal for H_2_SBM research with 461 publications, because this journal encourages the submission of original research, methodology papers and reviews relating to nitric oxide and other gasotransmitters such as H_2_S and carbon monoxide. The second mostly receptive journal in H_2_SBM research was FASEB Journal with 200 publications, followed by Antioxidants Redox Signaling with 116 publications and Free Radical Biology and Medicine with 115. The coverage of the last 2 journals clearly includes all gasotransmitters and relevant antioxidant signaling molecules. The 5th to 10th most prolific journals in publishing H_2_SBM research was Journal of Pharmacological Sciences (92), British Journal of Pharmacology (81), Journal of Biological Chemistry (70), Biochemical and Biophysical Research Communications (69), Gastroenterology (59), and Biochemistry (59).

### 3.4. The Most Prolific Countries with H_2_SBM Research Publications

**Figure 3:**
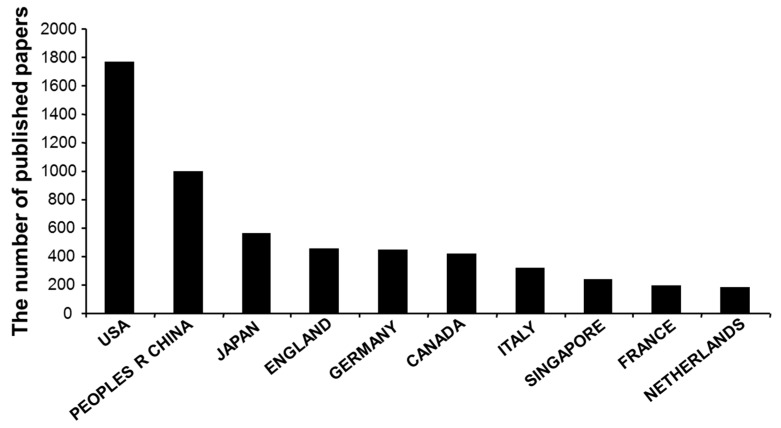
The most prolific countries with H_2_SBM research publications.

The publication indicators for the top 10 most prolific countries regarding H_2_SBM research were presented in Figure 3. The country with the greatest number of scientific publications in H_2_SBM research field was the USA with 1765 (30.0%) publications, followed by China with 995 (16.9%) publications, Japan with 555 publications (9.4%), England with 449 (7.6%) publications, and Germany with 444 (7.5%) publications. The 6th to 10th productive countries were Canada (413, 7.0%), Italy (314, 5.3%), Singapore (237, 4.0%), Netherlands (191, 3.2%), and France (178, 3.0%). The top 10 prolific countries account for 94.2% of the total number of scientific publications.

### 3.5. The Most Prolific Institutions with H_2_SBM Research Publications

Table 2 showed the top 10 productive institutes ranked by the numbers of publications. National University of Singapore was ranked the first in terms of publication output with 235 publications, followed by Peking University in China with 132 publications, University of Groningen in Netherlands with 114 publications, Fudan University in China with 113 publications, University of Exeter in England with 109 publications, and both Lakehead University (Canada) and University of Californian System (USA) with 104 publications. Among the top 10 institutions, 2 each were from China, England, and USA, one each in Canada, Japan, Netherlands, and Singapore. Based on the number of average citations per paper, National Center for Neurology Psychiatry (48.2), University of London (46.2), and Lakehead University (46.1) took the top 3 positions. National University of Singapore (55), Lakehead University (36), Peking University (33) and University of London (33) had the highest *h*-index.

### 3.6. The Support of H_2_SBM Research by Different Funding Agencies 

**Figure 4:**
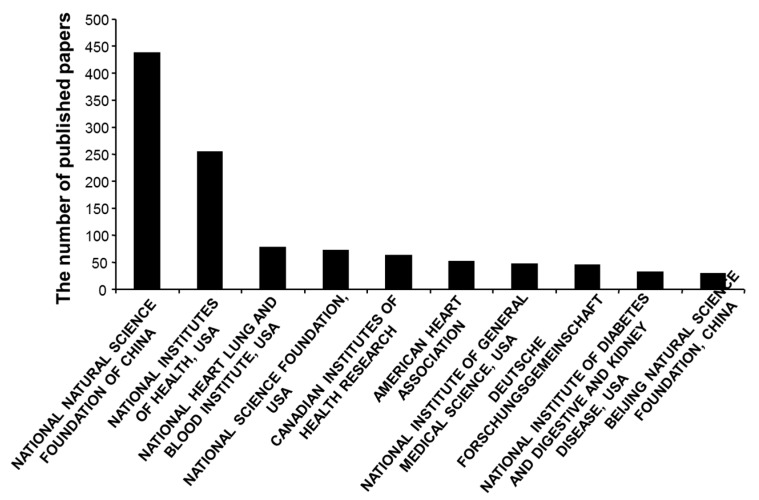
The support of H_2_SBM research by different funding agencies.

Funding acknowledgements from publications showed that National Natural Science Foundation of China supported the most H_2_SBM research with 437 (7.4%) publications, followed by the National Institute of Health of USA with 253 (4.3%) and National Heart Lung and Blood Institute of USA (76, 1.3%) (Figure 4). The fourth to tenth funding agencies in supporting H_2_SBM research were National Science Foundation of USA (71, 1.2%), Canadian Institute of Health Research (62, 1.1%), American Heart Association (51, 0.9%), National Institute of General Medical Sciences of USA (46, 0.8%), Deutsche Forschungsgemeinschaft (44, 0.7%), National Institute of Diabetes and Digestive and Kidney Diseases of USA (33, 0.6%), and Beijing Natural Science Foundation (28, 0.5%).

### 3.7. Researcher Citation Impact

The top 10 most prolific researchers in H_2_SBM publications are listed in Table 3. Among these 10 researchers, R. Wang from Canada had the highest citation being 8545 from 130 publications. The 2nd and 3rd most cited authors were PK Moore (7765) and H Kimura (6873) both with 115 publications. M Whiteman from England was the most prolific scientist with 136 publication cited by 3554 times. For these top 10 most productive scientists, PK Moore had the highest *h*-index of 47 and R Wang was the second with an *h*-index of 45, separating from the others with a gap. These eminent researchers were definitely considered to be the forerunners in H_2_SBM research.

### 3.8. Other Publication Performance Indicators

**Figure 5:**
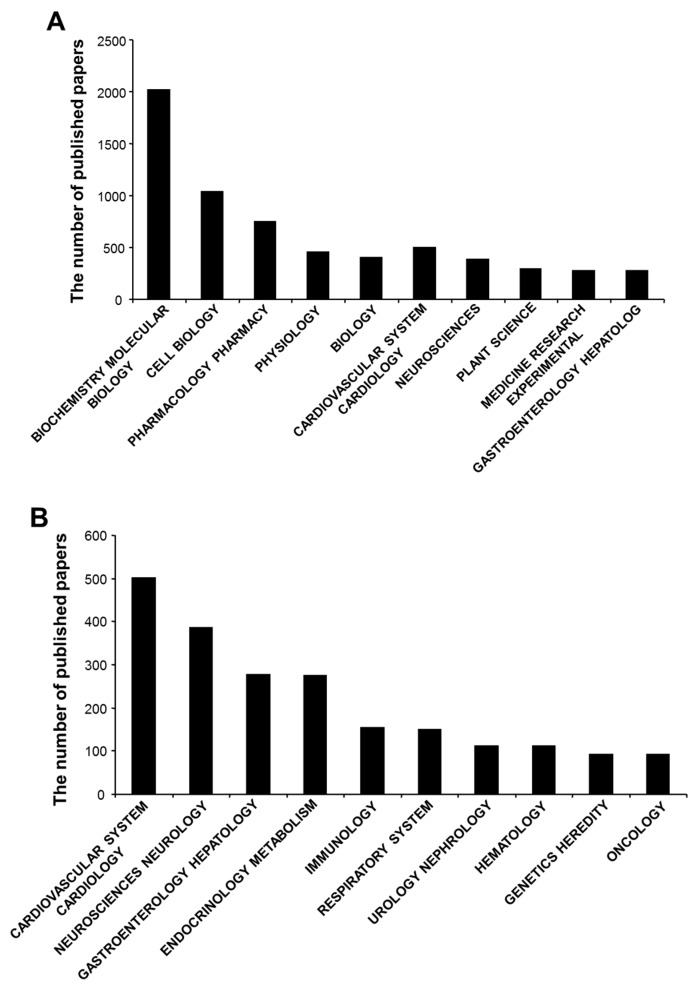
The mostly studied disciplines in H_2_SBM research. (**A**) the top 10 hottest disciplines in H_2_SBM research; (**B**) The top 10 hottest topics in H_2_S medicine research.

In general, the top 3 most extensively studied disciplines in H_2_SBM research were biochemistry molecular biology (34.3%), cell biology (17.7%), and pharmacology pharmacy (12.7%) (Figure 5A). Especially for H_2_S-related medicine research, cardiovascular system (8.5%), neuroscience (6.5%), and gastroenterology hepatology (4.7%) took the top 3 positions (Figure 5B). Furthermore, it was shown that 10.0% of the publications are review paper, while research articles contribute to 62.7% of the total publications (Table 4). The rest types of H_2_SBM publication were meeting abstract (23.0%), proceeding paper (3.0%), and others (1.1%). The average citations (42.5) for review papers were 64.1% more than that of research papers (25.9). In contrast, the *h*-index for research papers (120) was about 44.6% more than that of review papers (83). In these 5881 publications, only 6.2% of them were published in open-access journals (Table 5). The average citations per item (21.7) and *h*-index (136) in no open-access journals were much higher than those in open-access journals (9.4 per item and *h*-index of 27).

## 4. Discussion

Using bibliometrics to analyze H_2_SBM literatures allows us to uncover trends in the historical development and help us develop an understanding of the prevalent areas of interest in this field. To the best of knowledge, this study is the first of its kind to assess the productivity in the field of H_2_SBM during the period between 1990 and 2016 at global level.

Analysis of major research focuses indicated that cardiovascular function is the most common topic in H_2_S medicine research. Four of the top 10 mostly cited H_2_SBM papers explored the regulatory roles of H_2_S in vascular system. For example, some of these studies established the role of H_2_S as an endogenous K_ATP_ channel opener in vascular cells and the cardio-protective effects of H_2_S under both health and disease conditions [1,2,4,8]. Other hot topics included neuroscience and gastroenterology hepatology, etc. The research area of *S*-sulfhydration modification of protein and its impact on cellular functions is growing at the fastest pace (138 papers) since the first research paper on *S*-sulfhydration was published in 2009 [20], which provides the clue for the molecule-to-molecule interaction mechanism for the cellular functions of H_2_S. It should be noted that H_2_SBM research is also facing challenges in selective areas. Clinical trials comprised less than 3% of all publications, and phase I–III clinical trials were relatively scarce. The unstable chemical features and tissue-specific effects of H_2_S lead to the difficulty of developing druggable H_2_S-releasing compounds. There are strong demands for future application of H_2_S-based therapy.

In term of the number of published papers in H_2_SBM research, USA took the first place. The increasingly leading position of USA was also supported by the fact that two of the ten most productive institutions in H_2_SBM research were located in USA, including University of California System and University of Texas Medical Branch Galveston. China’s total expenditure on Research and Development has been significantly increased over the last 10 years, and National Natural Science Foundation of China was the most acknowledged funding agency in H_2_SBM publications. As a consequence, the total number of H_2_SBM papers from China was the second most among all countries, and two universities from China (Peking University and Fudan University) took the second and fourth position in the top 10 most prolific institutes. Singapore is the eighth most productive country in H_2_SBM research, while National University of Singapore is the number 1 most prolific institute in H_2_SBM research, attributable to the institution’s effort to assemble a H_2_S-focused pharmacology research unit and hiring a great number of H_2_SBM researchers in this university more than a decade ago. The ranks for the most prolific institutes or countries did not consider the trans-national or trans-institutional relocation of the leading H_2_SBM research team(s).

The number of citations an article received is a good indicator for its impact and contribution to research community. Based on the Web of Science, the total citations (7821) of the top 10 most cited papers count for 6.3% of all citations generated by 5881 H_2_SBM literatures, which reflects the importance of these papers. Nine from the 10 most highly cited articles were published in relatively higher–impact factor journals (IF > 5) except one from Biochemical and Biophysical Research Communications (IF = 2.47). The *h*-index, the number of published papers (N) that have been cited N or more times, is often used to assess the career-long citation impact of researchers [21,22]. One important finding was that PK Moore and R Wang had the highest *h*-index being 47 and 45 following a wide gap with other researchers in H_2_SBM research, reflecting their career-long important contribution and impact to the field. 

It is noted that the ratio of review papers to research articles was about 1:6.2. This trend needs to be carefully assessed. In general, review papers generate higher citations than original research articles (Table 4). This fact partially stimulates the interests of certain journals and researchers to produce review papers more than focusing on original research articles. We have seen too many review articles repeating the same topics by the same or different authors without new data or new ideas. We have seen review articles that review other review articles. This is the time to call the attention for H_2_SBM researchers and also all other biomedical researchers to produce more original research articles and lesser redundant reviews.

Although open access journals provide easy access to the published papers, no evidence suggests that open access articles receive significantly more citations than non-open access articles [23]. This is the same situation as H_2_SBM papers. Only a small portion of H_2_SBM (6.1%) was published in open-access journals, and the average citation and *h*-index for open-access journal paper were much lower than that from non-open access journal papers. On the other hand, the concept of open access journals is relatively new and the citation impacts of newly published papers require some time to manifest themselves. Based on the data from Web of Science, the journal of Oxidative Medicine and Cellular Longevity was the highest prolific open-access journal in H_2_SBM research with 44 publications since 2012, while the average citation per paper was only 4.16 dated on 15 October 2017. Scientific Reports, a new online open access journal, published 72 H_2_SBM articles from 2012 to 2016 (http://www.nature.com/srep/). However, this information could not be found in Web of Science.

In the bibliometric analysis completed, 98.6% of the 5881 publications found were published in English. This is not surprising since the majority of journals registered in Web of Science publish in English. In the top 10 countries that published the largest number of H_2_SBM, 4 countries are English-speaking, including USA, England, Canada, and Singapore, and total 48.7% publications were from these 4 countries. Of the 10 institutions that had the largest number of H_2_SBM publications, 6 were based in English-speaking countries. 

The readers are reminded of the limitations of this study. Only publications indexed by Web of Science were analyzed. It is known that only a small part of existing journals were indexed in Web of Science. Certainly, some H_2_SBM publications are missed or not included in this analysis. This study did not look at the most cited institutes due to technique difficulty and time limitation. It is worth noting that University of Saskatchewan from Canada contributed 32 publications in H_2_SBM research, while its average citation per item (152.28) was more than 3 times of those from the top 10 most prolific institutes (Table 2), clearly pointing to a pioneer position of University of Saskatchewan in H_2_SBM research. Another limitation is that, besides the categories of article and review, other document categories (e.g., meeting abstracts, editorial materials, letters, and notes, etc.) were also included in this study, which usually did not give sufficient study details. 

In conclusion, bibliometric analyses were performed to evaluate publication outcome related to H_2_SBM research at global level. An overall increase in H_2_SBM publications in the recent decades reflects the rapid advancement of this field. In the years to come there will be a continued increase in the productions of H_2_SBM research, probably with a similar trend observed in recent decades.

## Figures and Tables

**Table 1:** Top 10 mostly cited publications in H_2_SBM research.

Rank	Title	Author (First and/or Last Author)	Journal	Journal Impact Factor (2016)	Publishing Information	Citation (A)	Citation (B)	Citation (C)
1	The vasorelaxant effect of H_2_S as a novel endogenous gaseous K_ATP_ channel opener	Zhao W., Zhang J., Lu Y., Wang R. *	EMBO JOURNAL	9.792	1 November 2001;20(21):6008–6016.	1414	1073	1754
2	Two’s company, three’s a crowd: can H_2_S be the third endogenous gaseous transmitter?	Wang R. *	FASEB JOURNAL	5.498	November 2002;16(13):1792–1798.	1348	1025	1671
3	The possible role of hydrogen sulfide as an endogenous neuromodulator	Abe K., Kimura H. *	JOURNAL OF NEUROSCIENCE	5.988	1 February 1996;16(3):1066–1071.	1276	1016	1536
4	H_2_S as a physiologic vasorelaxant: hypertension in mice with deletion of cystathionine γ-lyase	Yang G., Wu L. *, Jiang B., Yang W., Qi J., Cao K., Meng Q., Mustafa A.K., Mu W., Zhang S., Snyder S.H. *, Wang R. *	SCIENCE	37.205	24 October 2008;322(5901):587–590.	1254	1061	1447
5	Hydrogen sulphide and its therapeutic potential	Szabó C. *	NATURE REVIEWS DRUG DISCOVERY	57.0	November 2007;6(11):917–935.	1038	866	1210
6	The possible role of hydrogen sulfide as an endogenous smooth muscle relaxant in synergy with nitric oxide	Hosoki R., Matsuki N., Kimura H. *	BIOCHEMICAL AND BIOPHYSICAL RESEARCH COMMUNICATIONS	2.466	28 August 1997;237(3):527–531.	901	693	1109
7	Hydrogen sulfide attenuates myocardial ischemia-reperfusion injury by preservation of mitochondrial function	Elrod J.W., Calvert J.W., Morrison J., Doeller J.E., Kraus D.W., Tao L., Jiao X., Scalia R., Kiss L., Szabo C., Kimura H., Chow C.W., Lefer D.J. *	PROCEEDINGS OF THE NATIONAL ACADEMY OF SCIENCES OF THE UNITED STATES OF AMERICA	9.661	25 September 2007;104(39):15560–15565.	675	559	791
8	Physiological implications of hydrogen sulfide: a whiff exploration that blossomed	Wang R. *	PHYSIOLOGICAL REVIEWS	27.312	April 2012;92(2):791–896.	634	537	731
9	Hydrogen sulfide and cell signaling	Li L., Rose P., Moore P.K. *	ANNUAL REVIEW OF PHARMACOLOGY AND TOXICOLOGY	12.877	2011;51:169–187.	616	535	697
10	Hydrogen sulfide protects neurons from oxidative stress	Kimura Y., Kimura H. *	FASEB JOURNAL	5.498	July 2004;18(10):1165–1167.	597	456	738

Note 1: The rank of this table was based on Citation A, which is the average of Citation B and Citation C. Citation B was based on Web of Science Core Collection. Citation C was based on Google Scholar. * Corresponding author. Note 2: The data summarized in this table were collected on 15 October 2017.

**Table 2:** The top 10 most productive institutes in H_2_SBM research.

Rank	Institute	Total Publication	Total Citations	Average Citations per Item	*h*-Index
1	National University of Singapore	235	9424	40.1	55
2	Peking University, China	132	3564	27.0	33
3	University of Groningen, Netherlands	114	1889	16.6	26
4	Fudan University, China	113	2633	23.3	28
5	University of Exeter, England	109	1793	16.5	21
6	Lakehead University, Canada	104	4791	46.1	36
7	University of California System, USA	104	2660	25.6	28
8	University of Texas Medical Branch Galveston, USA	103	1654	25.0	25
9	National Center for Neurology Psychiatry, Japan	102	4911	48.2	31
10	University of London, England	96	4432	46.2	33

Note: The rank of this table was based on the number of total publications.

**Table 3:** The top 10 most cited authors in H_2_SBM research.

Rank	Researcher	Total Citations	Total Publication	Average Citations per Item	*h*-Index
1	Wang R.	8545	130	65.73	45
2	Moore P.K.	7765	115	67.52	47
3	Kimura H.	6873	115	59.77	35
4	Whiteman W.	3554	136	26.13	28
5	Wallace J.L.	3163	69	45.84	26
6	Tang C.S.	2936	89	32.99	30
7	Bian J.S.	2833	75	37.77	30
8	Szabo C.	2794	120	23.28	28
9	Du J.B.	2602	81	32.12	29
10	Kawabata A.	1058	78	13.56	17

Note: The rank of this table was based on the number of total citations. Based on the Web of Science data of the top 10 most prolific scientists in H_2_SBM research for the period of 1990–2017 (dated on 15 October 2017), the total citations for each researcher was collected and ranked.

**Table 4:** The publication and citation impacts of original research papers, abstract, and review papers in H_2_SBM research.

Type	Total Publication	Total Citations	Average Citations per Item	*h*-Index
Research	3690	95,542	25.9	120
Abstract	1355	190	0.1	4
Review	591	25,085	42.5	83

**Table 5:** Number of H_2_SBM papers published in non-open access and open access journals.

Open Access	Number of Published Papers	Total Citation	Average Citation per Item	*h*-Index
NO	5519	119,657	21.7	136
YES	362	3417	9.4	27
